# Suppression of Neutrophil Antimicrobial Functions by Total Particulate Matter From Cigarette Smoke

**DOI:** 10.3389/fimmu.2018.02274

**Published:** 2018-10-04

**Authors:** Yao Zhang, Shuo Geng, G. L. Prasad, Liwu Li

**Affiliations:** ^1^Department of Biological Sciences, Virginia Tech, Blacksburg, VA, United States; ^2^RAI Services Company, Winston-Salem, NC, United States

**Keywords:** neutrophil programming, TPM, immune-modulation, NADPH oxidase, inflammation

## Abstract

Chronic cigarette smoking is widely known to alter immune functions and compromise host defense against microbial infection. Neutrophils play an essential role in the immune defense against microbial pathogens and also participate in the development of the inflammatory responses. However, there is limited information about the effects of cigarette smoking on neutrophil response. In this study, cultured bone marrow neutrophils were exposed to total particulate matter (TPM) from cigarette smoke. We found that TPM not only reduced LPS-induced TNFα production, but also suppressed neutrophil bactericidal activity. We also observed that TPM priming reduced the expression of NADPH oxidase component gp91 and iNOS, molecules important for bacterial killing. Mechanistically, we documented that TPM-primed neutrophils have reduced STAT1 activation following subsequent LPS challenge. STAT1 is a key transcription factor responsible for the expression of inflammatory genes as well as gp91 and iNOS. Collectively, reduced STAT1 activation and reduced NADPH oxidase/iNOS may potentially explain the compromised anti-microbial function of TPM-programmed neutrophils. Taken together, our findings reveal that the key innate immune neutrophil is subject to reprogramming by smoking to adopt an immune-suppressed state, potentially responsible for chronic smoking-mediated immunosuppression.

## Introduction

Cigarette smoking has been well recognized as a major risk factor for lung diseases such as chronic obstructive pulmonary disease (COPD), pneumonia, asthma, and lung cancer (1, 2). The pathogenesis of smoking-associated lung diseases is highly complex and potentially involves altered innate and adaptive immune responses. Alterations in adaptive immunity and subsequent inflammatory complications may underlie chronic airway remodeling associated with smoking-mediated COPD and cancer (3–5). In addition, smoking is also reported to directly affect the function of innate immune cells such as macrophages, dendritic cells, natural killer cells, and neutrophils, potentially contributing to compromised innate defense and execerbated lung infection (6–10).

Although past studies provided compelling evidence with regard to smoking-related alteration of adaptive immune cells, relatively few mechanistic studies are available in the context of altered innate immune cell functions related with smoking. Alveolar macrophages from cigarette smokers exhibit reduced phagocytic activity toward airway pathogens (11–13). In addition, compromised dendritic cells and natural killer cells may also collectively contribute to compromised bacterial killing function (7, 14). Alterations in neutrophil functions in smokers have been largely associated with increased inflammation and tissue damage (15, 16). Cigarette smoke-activated neutrophils from smokers congregate in lung vasculature, causing elevated release of inflammatory mediators such as metalloproteinase-9 (17) and Leukotriene B4 (LTB4) (18, 19). These mediators subsequently cause oxidative and proteolytic tissue damage, aggravating immune dysregulation leading to chronic lung disease. However, it is less clear whether cigarette smoking or exposure to cigarette smoke may directly impair the bacterial-killing function of neutrophils.

To address this question, we hereby examined whether neutrophil-mediated bacterial killing is impacted by total particulate matter (TPM) from cigarette smoke. We have compared the bacterial killing potential of naïve and TPM-challenged primary murine neutrophils and have investigated underlying mechanisms. We report that TPM treatment significantly compromises bacterial killing activity of neutrophils. Mechanistically, NADPH oxidase as well as inducible nitric oxide synthase (iNOS) are important in mediating the bacterial-killing activity. We observed that TPM-treated neutrophils fail to induce gp90 and iNOS expression following LPS challenge. STAT1 is a critical transcription factor involved in the expression of both gp90 and iNOS (20, 21). We observed that TPM treatment compromises the STAT1 activation induced by LPS. Taken together, our study reveals novel mechanisms for TPM-mediated neutrophil reprogramming with reduced bacterial killing activity, in that TPM reduces STAT1 mediated expression of key anti-bacterial molecules such as gp90 and iNOS.

## Materials and methods

### Mice

Wild type (WT) C57BL/6 mice were purchased from the Charles River laboratory. All mice were housed under specific pathogen-free conditions and bred and maintained in the animal facility at Virginia Tech with the approved Animal Care and Use Committee protocol. Groups of 6–8 weeks old mice were used in all experiments.

### Reagents

LPS (*Escherichia coli* 0111:B4) was obtained from Sigma Aldrich. TPM from 3R4F reference cigarettes was prepared as described previously and provided by R.J. Reynolds Tobacco Company, Winston-Salem, NC (22). FITC conjugated anti-mouse Ly6G antibody, PE/Cy7 conjugated anti-mouse TNFa antibody, APC/Cy7 and PE conjugated anti-mouse/human CD11b antibody, were from Biolegend (San Diego, CA).

### TPM preparation

TPM from 3R4F reference cigarettes (University of Kentucky) was prepared at Labstat International, Kitchner, Canada as described previously (22). The reference cigarettes were smoked using the standard ISO method (35-60-2, puff volume in mL, inter puff interval in seconds and puff duration in seconds). The particular matter was collected on a Cambridge filter pad and dissolved in DMSO. The nicotine content of the TPM was quantified, and expressed in μg/mL. We used this measure, termed equi-nicotine units, to compare the effect of TPM and to normalize the results.

### Neutrophil isolation and culture

**Figure 1:**
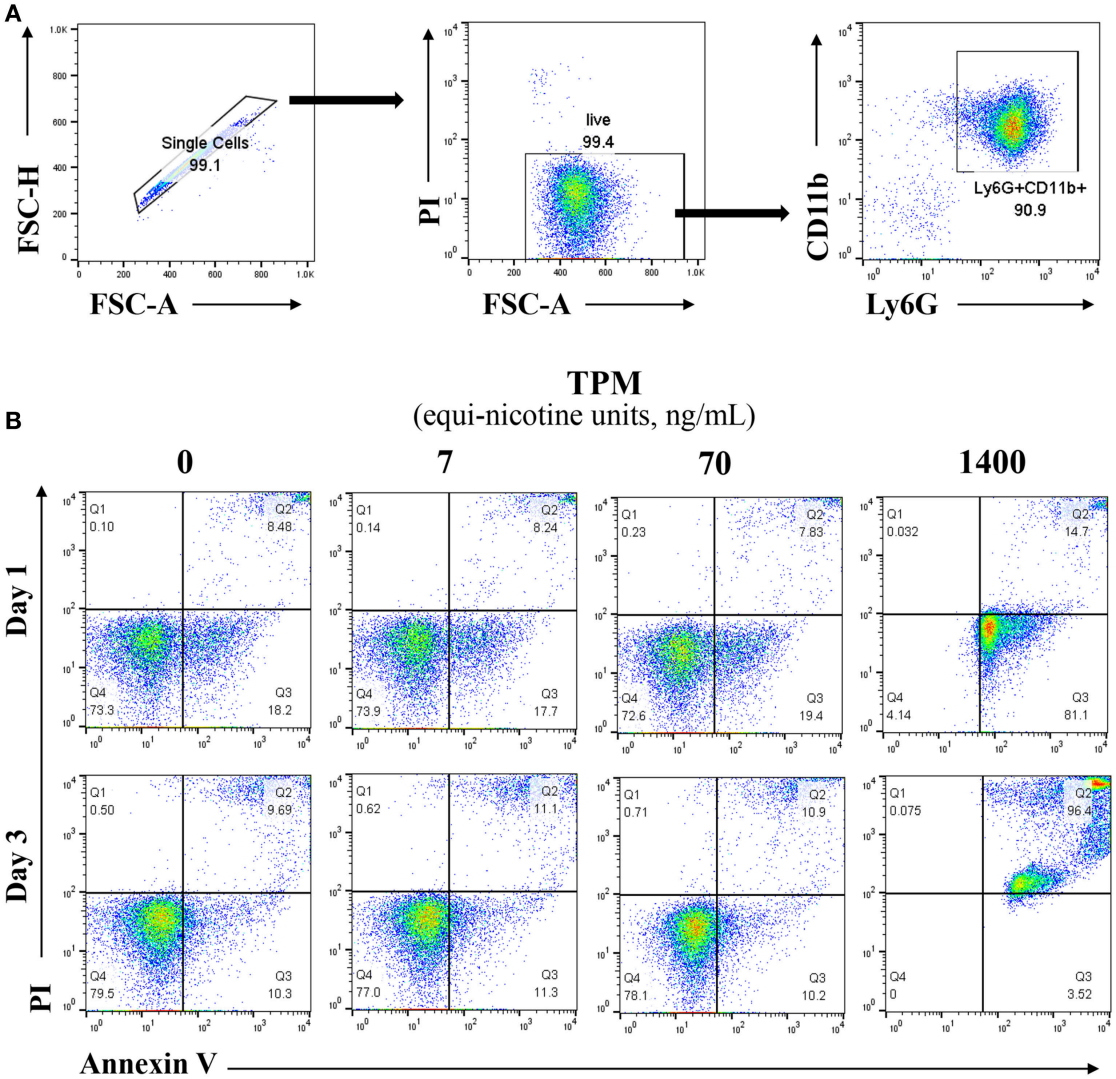
Lower dosages of TPM do not increase neutrophil cell death. **(A)** Reprensentive flow cytometry analysis of viability and purity of isolated neutrophils from mouse bone marrow through percoll gradient as described in the Materials and Methods section. **(B)** Purified neutrophils were cultured in completed RPMI medium. Neutrophils were treated with different concentrations (ng/ml equi-nicotine units) of TPM for 3 days. Flow cytometry analyses were performed to measure cell death as described in the Materials and Methods section. Representative results from three experiments are shown. Numbers on dot plots represent the percentage of apoptotic neutrophils cells stained by PI and annexin V.

Neutrophils from bone marrow were isolated from the tibias and femurs of wild type (WT) mice, purified over a Percoll gradient of 82, 65, and 55%. Neutrophils were collected at the 65–82% interface, and cultured in completed RPMI (10% FBS, 10 mM HEPES, 1% penicillin/streptomycin, L-glutamine 2 mM, and 10 ng/ml G-CSF) (23). The purity and viability of neutrophils post purification and before the start of the experiment were >90% as measured by flow cytometry (Figure 1A).

### Flow cytometry

Single-cell suspensions were prepared from bone marrow (23). Prior to intracellular staining for cytokines, cells were cultured *in vitro* for 4 h in RPMI completed medium with GolgiStop^TM^ (BD Biosciences, San Jose, CA). Then the cells were fixed after surface marker staining, permeabilized and stained with anti-TNFa, according to the manufacturer's instructions (BD Biosciences). Samples were analyzed with a FACS Canto II (BD Biosciences). FACS plots shown were analyzed with FlowJo (Ashland, OR).

### *In vitro* bacterial killing assay

Fresh overnight cultures of *E.coli* were resuspended in HBSS and opsonized with 20% mouse serum. Purified neutrophils were cultured and treated with LPS (100 ng/ml) or TPM (70 ng/ml of equi-nicotine units) overnight. The primed neutrophils and opsonized bacteria were incubated together at 1:5 ratio for 1 h. Following the incubation, neutrophils were lysed by 0.05% triton and diluted aliquots were spread on an LB plate. Bacterial colonies were counted the next day (24). Bacterial suspension without any cells were used as input control. Relative killing rates were calculated by (input CFU-leftover CFU)/input CFU.

### NADPH measurement

Purified neutrophils were cultured and treated with LPS (100 ng/ml) and/or TPM (70 ng/ml equi-nicotine units) overnight, followed with or without stimulation of PMA (20 ng/ml) for 15 min, then subjected to NADP/NADPH-Glo^TM^ assay, according to the manufacturer's instructions (Promega, Madison, WI).

### Protein extraction and western blot analyses

Cells were washed with PBS and harvested in 1 × SDS lysis buffer containing protease inhibitor cocktail and subjected to SDS-PAGE (25). Seperated proteins were transferred to an immunoblot PVDF membrane (BioRad). Western blot analyses were performed with specified antibodies as shown in reagents above.

### ELISA

Purified neutrophils were pre-treated without or with autophagy inhibitor Spautin1 30 min before stimulation with LPS (100 ng/ml) and/or TPM (70 ng/ml equi-nicotine units) overnight, then conditional medium was collected. TNFα levels in the conditional medium were analyzed by enzyme-linked immunosorbent assay (ELISA) using ELISA Kit from eBioscience according to the manufacturer's instructions.

### Confocal microscopy

Neutrophils were treated with PBS, LPS, or TPM overnight, and then fixed with 4% PFA, deposited on slides through cytospin, and permeabilized with 0.2% Trinton X-100. The cells were blocked and stained with Alexa Fluor 488 anti-COX4 (NOVUS) antibody and Cy3-anti-LAMP1 antibody (Abcam) in the dark at room temperature. The samples were observed under a confocal microscope.

### Statistics

All experiments were performed at least 3 times. Representative and reproducible results were shown. Statistical analysis was performed with Prism software (GraphPad Software, La Jolla, CA). Values were expressed as means ± SEM. The significance of the differences was assessed by Student's *t*-test. *P* < 0.05 was considered statistically significant.

## Results

### Dose selection of total particulate matter (TPM) for neutrophil survival

In order to optimize our *in vitro* experimental system for evaluating neutrophil responses to TPM challenge, we first tested the effects of varying dosages of TPM on neutrophil survival *in vitro*. As shown in Figure 1B, compared to vehicle (DMSO) treatment, higher dose of TPM (1.4μg/ml equi-nicotine units) resulted in 99% neutrophils undergoing apoptosis after 1 day culture, suggesting that high dose of TPM (1.4 μg/ml equi-nicotine units) is toxic to neutrophils. However, neutrophil survival within both 1 and 3 day culture periods under the treatment of relatively lower doses of TPM (7 or 70 ng/ml equi-nicotine units) were comparable to neutrophils treated with vehicle control. This nanogram-range dosage may also reflect dosages experienced by human smokers (26). Upon optimization of TPM dosages, the non-toxic dose (70 ng/ml of equi-nicotine units) of TPM that enabled cell survival was chosen for further examination of neutrophil functional studies.

### TPM suppresses LPS-induced TNFα production

**Figure 2:**
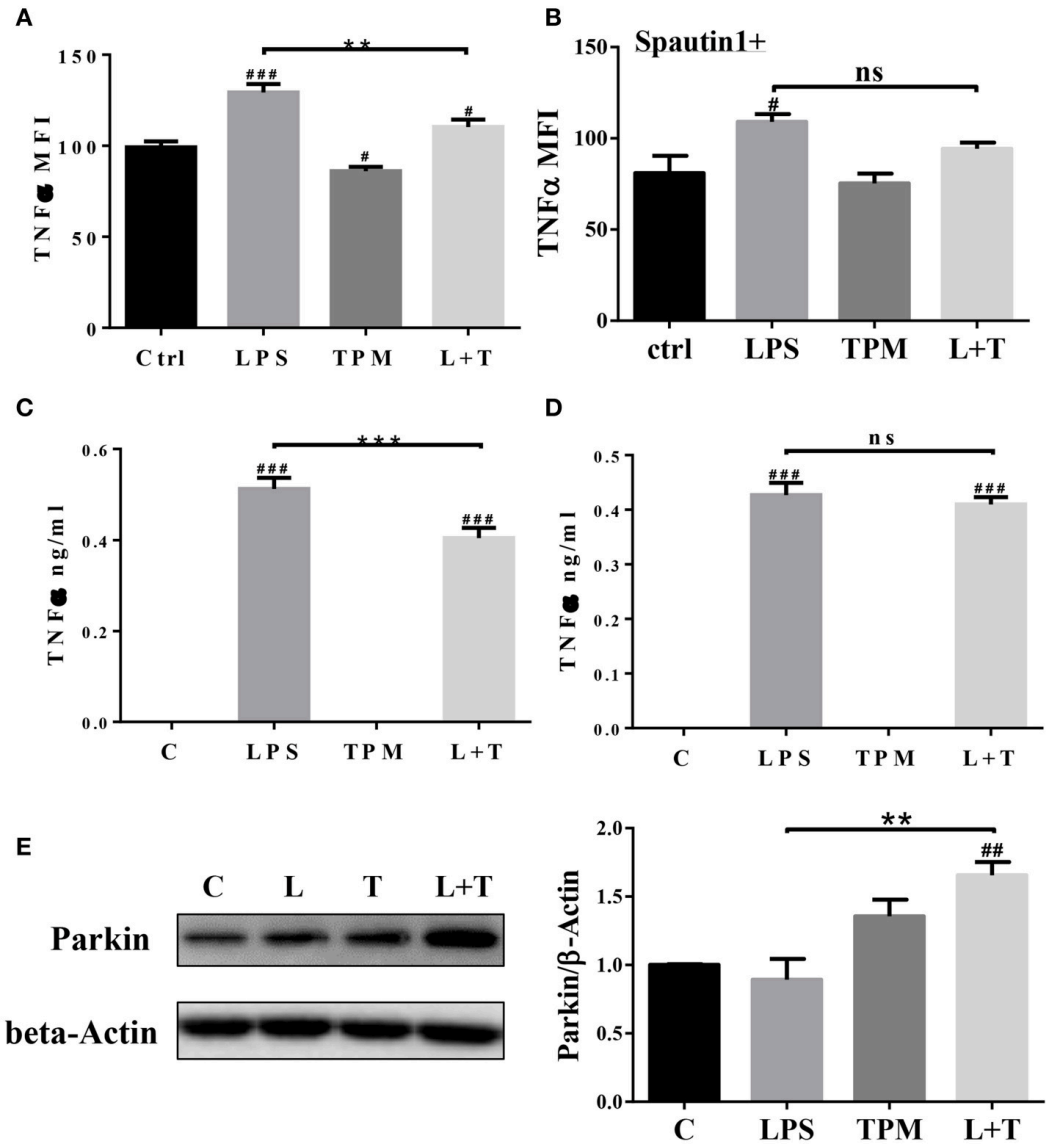
TPM reduced TNFα production by neutrophils induced by LPS. Bone marrow neutrophils were pre-treated without **(A)** or with autophagy inhibitor Spautin1 **(B)** 30 min before stimulation with LPS (1μg/ml) and/or TPM (70 ng/ml equi-nicotine units) for 4 h. Intracellular TNFα levels were measured by flow cytometry analysis. **p < 0.01; ^#^*p* < 0.05, ^*###*^*p* < 0.001 as compared with control group. Purified neutrophils were pre-treated without **(C)** or with autophagy inhibitor Spautin1. ***p < 0.001; ^*###*^p < 0.001 as compared with control group. **(D)** 30 min before stimulation with LPS (100 ng/ml) and/or TPM (70 ng/ml equi-nicotine units) overnight, then conditional medium was collected and subjected to ELISA analysis. **(E)** Purified neutrophils were treated with LPS (100 ng/ml) and TPM (70 ng/ml equi-nicotine units) overnight, then subjected to immunoblotting. Bar graph on the right panel is the quantification results of protein expression from three experiments, normalized to β-actin. **p < 0.01; ^*##*^*p* < 0.01 as compared with control group.

In response to inflammatory signals such as lipopolysaccharide (LPS), neutrophils produce cytokines and chemokines, which play key roles in the regulation of the immune response and mount an effective anti-microbial defense (27). We next tested whether low-dose TPM exposure may affect the expression of selected inflammatory cytokines by neutrophils in response to LPS. To test this, neutrophils were treated with 1 μg/ml LPS, in the presence or absence of TPM, for 4 h as described in Materials and Methods. Treated cells were stained for intracellular TNFα. As a positive control, we observed that LPS treatment alone increased TNFα levels produced by neutrophils (Figures 2A,C). TPM alone also reduced TNFα expression in neutrophils. Furthermore, when neutrophils were co-stimulated with TPM and LPS, the levels of TNFα were significantly reduced as compared to cells treated with LPS alone (Figures 2A,**C**). Our data suggest that TPM is a negative regulator of neutrophil inflammatory activation.

To determine its potential mechanism, we tested whether TPM may attenuate neutrophil inflammatory activation through inducing homeostatic autophagy. Autophagy is involved in reducing cellular stress and is known to be activated by TPM (28). To test whether autophagy is involved in TPM-mediated suppression of TNFα expression, autophagy inhibitor, spautin-1, was added to the culture system. As shown in Figures 2B,D, inhibition of autophagy by spautin-1 blocked the TPM effect and restored TNFα induction by LPS in the presence of TPM.

**Figure 3:**
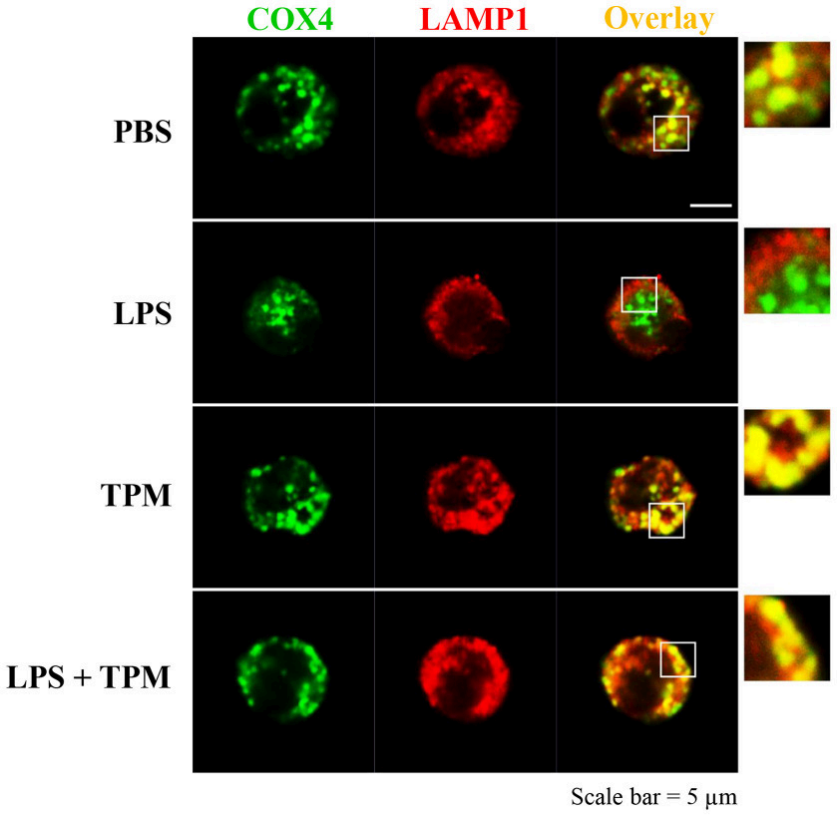
TPM restored mitophagy in neutrophils challenged with LPS. Bone marrow neutrophils were treated with either PBS, LPS (1μg/ml) and/or TPM (70 ng/ml equi-nicotine units) for 24 h. Cells were fixed and co-stained with fluorescently-conjugated anti-COX4 (mitochondria marker) antibody as well as anti-LAMP1 (lysosome marker) antibody. Cells were visualized by confocal microscopy.

We further measured the levels of Parkin, a molecule involved in the initiation of mitophagy, and observed an induction of Parkin by TPM and LPS co-treatment, indicative of mitophagy activation by TPM (Figure 2E). To further independently confirm that LPS-mediated mitophagy disruption can be remedied by TPM, we performed confocal analyses. Consistent with our Western blot analyses presented above, LPS challenge dramatically disrupted the fusion of mitochondria and lysosome. In contrast, TPM co-treatment restored mitophagy in cells challenged with LPS (Figure 3). Together, our data suggest that TPM reduces LPS-induced TNFα expression through inducing autophagy-mediated homeostasis.

### TPM decreases neutrophil bacterial killing activity

**Figure 4:**
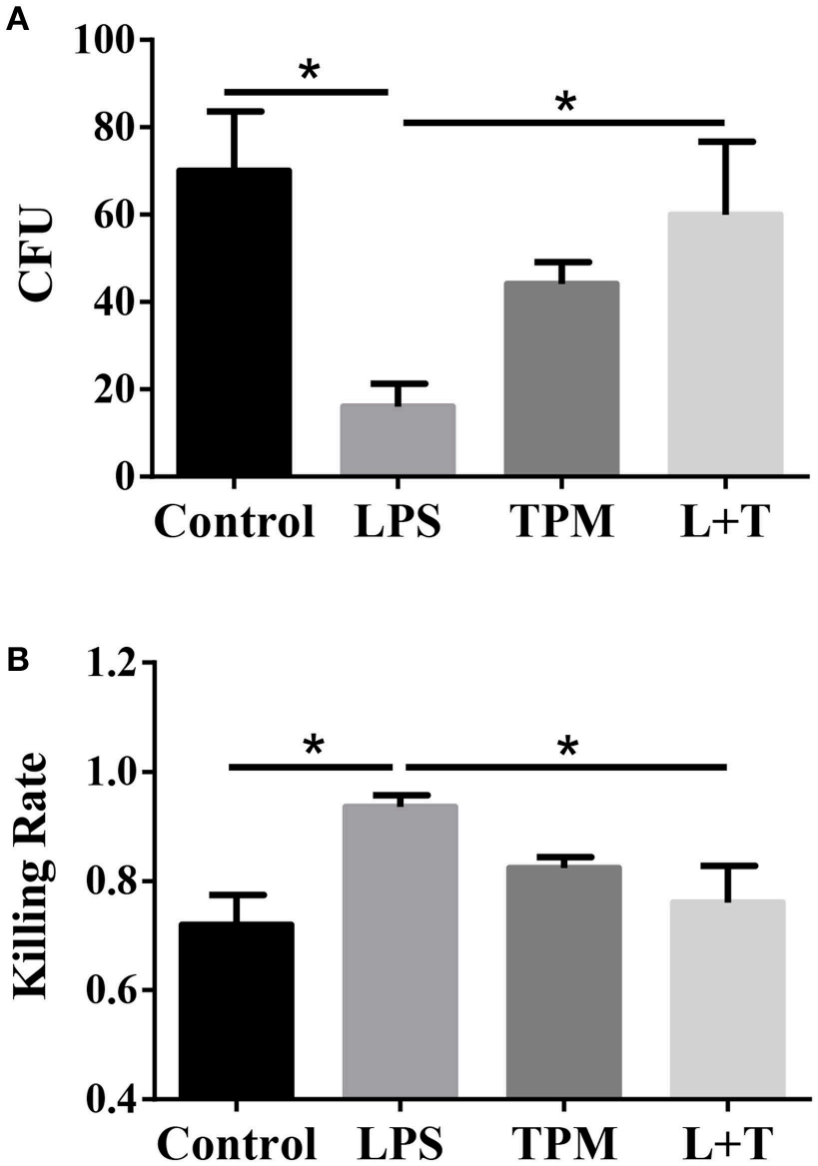
TPM decreased the bacteria killing activity of LPS-primed neutrophils. **(A)** *In vitro* bacterial killing by neutrophils primed with LPS and/or TPM. Intra-cellular bacteria harvested from neutrophils were plated on agar plates, incubated overnight, and quantified as bacteria colony-forming units (CFU). **(B)** Relative bacterial killing rates were calculated according to the input control. Data represent three experiments. *p < 0.05.

Elimination of invading microbes is one of the major functions of neutrophils. Since TPM potentially suppressed neutrophil activation in terms of TNF expression, we next studied whether TPM may also compromise the bacterial killing capability of neutrophils. To test this, neutrophils were treated with LPS and/or TPM overnight, then loaded with opsonized *E.coli* for 1 h, followed by cell lysis. Cell lysates were plated on bacterial growth media, and surviving bacteria colonies were counted the next day. As shown in Figure 4, LPS priming increased neutrophil bactericidal activity, as reflected by lower CFU counting (Figure 4A) and higher bacterial killing rate (Figure 4B). However, in cells pre-treated with both LPS and TPM, CFU counts were significantly elevated as compared to cells pre-treated with LPS alone. Our data show that TPM compromises the bactericidal activity of neutrophils.

### TPM reduces NADPH oxidase components and stat1 activation

**Figure 5:**
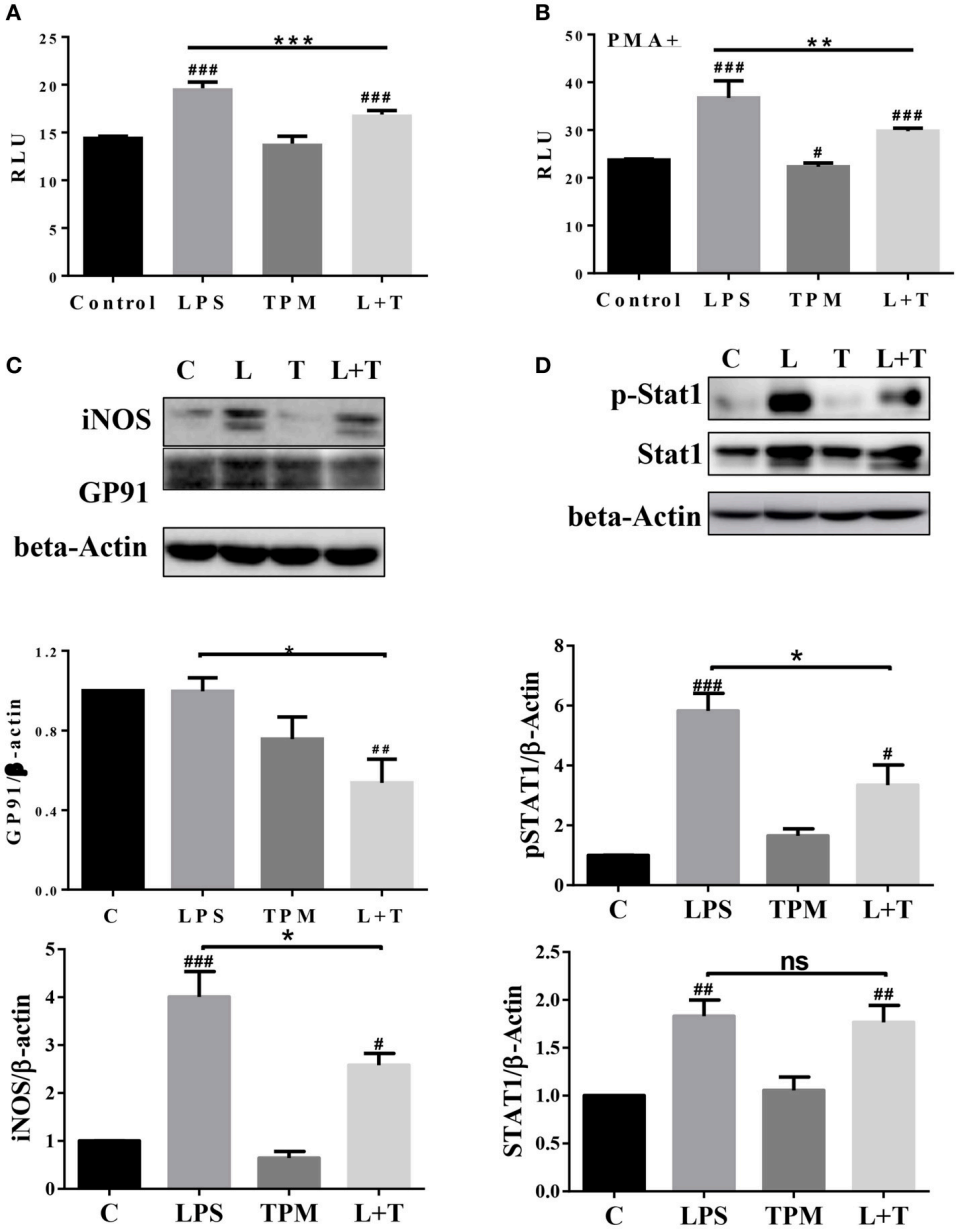
TPM decreased the levels of iNOS, NADPH oxidase component GP91, and STAT1 activation in LPS-primed neutrophils. Purified neutrophils were treated with LPS (100 ng/ml) and/or TPM (70 ng/ml equi-nicotine units) overnight, followed without **(A)** or with **(B)** stimulation of PMA (20 ng/ml) for 15 min, then subjected to NADP/NADPH measurement. **p < 0.01, ***p < 0.001; ^#^*p* < 0.05, ^*###*^*p* < 0.001 compared with control group. **(C,D)** Purified neutrophils were treated with LPS (100 ng/ml) and/or TPM (70 ng/ml equi-nicotine units) overnight, then subjected to immunoblotting to measure the levels of iNOS, gp91, total STAT1, and p-STAT1. Data represent three experiments. Bar graphs on the bottom panels are the quantification results of protein expressions, normalized to β-actin. *p < 0.05; ^#^*p* < 0.05, ^*##*^*p* < 0.01 compared with control group.

In order to further determine molecular mechanisms for TPM-mediated reduction of neutrophil bacterial killing activity, we tested neutrophil NADPH oxidase components involved in oxidative burst and bacterial killing. Neutrophils were treated with LPS and/or TPM for 24 h. Treated cells were subjected to the measurement of NADP/NADPH levels as described in the Materials and Methods section. The levels of NADP/NADPH were increased in neutrophils treated with LPS, and reduced in neutrophils subjected to the co-treatment with LPS and TPM (Figure 5A). Similar results were also observed in neutrophils primed with PMA (Figure 5B). We further examined the levels of NADPH oxidase component gp91 and observed a reduction of gp91 in neutrophils co-stimulated with LPS and TPM. In addition, we also observed that TPM treatment reduced the expression of iNOS induced by LPS (Figure 5C). Together, our data suggest that TPM is a negative regulator of NADPH oxidase in neutrophils and also reduces the expression of iNOS.

Next, we examined the potential molecular mechanisms that underlie the reduced neutrophil activation by TPM treatment. STAT1 has been shown to be critically involved in neutrophil activation and the expression of pro-inflammatory as well as anti-bacterial mediators such as iNOS and gp96 (29, 30). We therefore tested the activation status of STAT1 by probing the phosphorylated pSTAT1 levels in neutrophils treated with LPS and/or TPM. As shown in Figure 5D, LPS significantly induced the pSTAT1 levels in neutrophils. TPM alone has no effect on neutrophil pSTAT1 levels. Co-treatment with TPM and LPS attenuated the neutrophil pSTAT1 levels induced by LPS. Our data reveal that TPM may contribute to compromised neutrophil bacterial killing activity by reducing STAT1-mediated expression of NADPH oxidase and iNOS.

## Discussion

The results reported in this manuscript indicate that low-dose cigarette smoke TPM compromises the anti-microbial defense function of neutrophils. Several lines of evidence support our conclusion. First, we observed that neutrophils treated with low-dose TPM demonstrate reduced bacterial killing activity without apparent alteration in neutrophil viability. Second, we documented that TPM-treated neutrophils have reduced capacity to express inflammatory cytokines, reduced bacterial killing activity, reduced expression of iNOS, and reduced NADPH oxidase components. Third, TPM-treated neutrophils have reduced activation of STAT1, a key transcription factor involved in the expression of iNOS and key NADPH oxidase components such as gp91.

At the translational level, our data provide a conceptual advance in terms of how TPM may compromise host immune environment and contribute to chronic health conditions observed in smokers. Past studies regarding TPM on immune cells largely focused on the high dosages of TPM on cellular survival and death (22, 31). Although these studies are important and may reveal the highly toxic effect of smoking, they may not fully reflect the chronic effects of regular smoking with low-levels exposure. Our data reveal that low-levels of TPM exposure is sufficient to compromise neutrophil functions without apparent cell death. Neutrophils are predominant innate leukocytes in the host lung and related mucosal tissues. Proper functions of neutrophils are essential for maintaining proper host defense toward infectious and inflammatory signals (32). Compromised neutrophil anti-microbial function may subject the host to higher risks of chronic infection, as often seen in humans with chronic smoking (33, 34).

Our data also provide additional support for the emerging concept of innate immune programming and memory. Although previous dogma claim that innate leukocytes such as monocytes and neutrophils are first responders to challenges with limited modulatory roles nor memory, recent studies suggest that more complex programming and memory dynamics of neutrophils may exist (35). For example, tumor-infiltrating neutrophils tend to adopt an anti-inflammatory state with elevated expression of TGFβ (36). Humans with chronic alcohol consumption tend to have neutrophils with reduced anti-microbial functions (37). Consequently, reprogrammed neutrophils with altered functions may pre-dispose the host for the pathogenesis of chronic diseases. Our current data lend further support for this concept, and reveal that TPM from cigarette smoke may program neutrophils into an immune-compromised state with reduced anti-microbial capability. Our data lend mechanistic support for previous epidemiology observations that document increased risks for microbial infections in chronic smokers (38–40). Chronic smokers were known to exhibit elevated risks, severities with increased relapse, and mortality associated with tuberculosis (38). Both direct and second-hand smoke exposure are also reported to be associated with higher risks of lung microbial infections causing pneumococcus meningitis (39). Many of the adverse effects such as COPD related with chronic smoking may linger long after smoking cessation (40). Reprogrammed neutrophils with compromised anti-microbial activity due to TPM challenge as we reported herein may underlie elevated risks of chronic infection and lung pathology.

At the molecular level, our data reveal that TPM skews neutrophil function through reducing STAT1-mediated expression of inflammatory mediators as well as critical anti-bacterial components such as iNOS and NADPH oxidase components, both of which are important for the anti-bacterial function of neutrophils (41, 42). Our data also reveal that TPM may reduce neutrophil inflammatory activation through inducing mitophagy. Further mechanistic studies are warranted to dissect the detailed mechanisms that underlie the regulation of neutrophil homeostasis by TPM. Collectively, our study has revealed an important modulatory role of TPM in compromising the anti-bacterial killing function of neutrophils.

## Ethics statement

This study was carried out in accordance with the recommendations of Virginia Tech Institutional Guideline for proper usage of animals. The protocol was approved by the Institutional Animal Care and Usage Committee of Virginia Tech.

## Author contributions

YZ conducted the experiment, analyzed the data, and wrote the manuscript. SG conducted the experiment and analyzed the data. GP analyzed the data and wrote the manuscript. LL designed the study, analyzed the data, and wrote the manuscript.

### Conflict of interest statement

GP, is a full time employee of RAI Services Company. RAI Services Company is a wholly owned subsidiary of Reynolds American Inc., which is a wholly owned subsidiary of British American Tobacco plc. The remaining authors declare that the research was conducted in the absence of any commercial or financial relationships that could be construed as a potential conflict of interest.
